# Natural Presentation of Glycosaminoglycans in Synthetic Matrices for 3D Angiogenesis Models

**DOI:** 10.3389/fcell.2021.729670

**Published:** 2021-10-04

**Authors:** Cornelia Zapp, Patricia Mundinger, Heike Boehm

**Affiliations:** ^1^Department of Cellular Biophysics, Max Planck Institute for Medical Research, Heidelberg, Germany; ^2^Institute for Physical Chemistry, Heidelberg University, Heidelberg, Germany

**Keywords:** glycosaminoglycans, terminal thiolation of glycosaminoglycans, hyaluronan, hydrogels, thiol-Michael addition, angiogenesis

## Abstract

Glycosaminoglycans (GAGs) are long, linear polysaccharides that occur in the extracellular matrix of higher organisms and are either covalently attached to protein cores, as proteoglycans or in free form. Dependent on their chemical composition and structure, GAGs orchestrate a wide range of essential functions in tissue homeostasis. Accordingly, GAG-based biomaterials play a major role in tissue engineering. Current biomaterials exploit crosslinks between chemically modified GAG chains. Due to modifications along the GAG chains, they are limited in their GAG-protein interactions and accessibility to dissect the biochemical and biophysical properties that govern GAG functions. Herein, a natural presentation of GAGs is achieved by a terminal immobilization of GAGs to a polyethylene glycol (PEG) hydrogel. A physicochemical characterization showed that different end-thiolated GAGs can be incorporated within physiological concentration ranges, while the mechanical properties of the hydrogel are exclusively tunable by the PEG polymer concentration. The functional utility of this approach was illustrated in a 3D cell culture application. Immobilization of end-thiolated hyaluronan enhanced the formation of capillary-like sprouts originating from embedded endothelial cell spheroids. Taken together, the presented PEG/GAG hydrogels create a native microenvironment with fine-tunable mechanobiochemical properties and are an effective tool for studying and employing the bioactivity of GAGs.

## Graphical Abstract

The graphical abstract on mechanism of the hepatoprotective effect of cos against ALI.

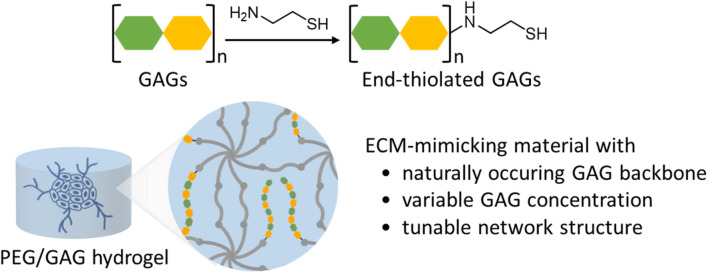

## Introduction

The mammalian extracellular matrix (ECM) is a complex and highly dynamic meshwork consisting of diverse bioactive macromolecules, such as fibrous structural proteins, glycosaminoglycans and adhesion mediating molecules, including glycoproteins and proteoglycans (Theocharis et al., 2016). The ECM provides structural and functional support for the cells, although the exact composition differs tremendously among tissues within the body. Resident cells constantly synthesize, degrade and remodel the extracellular molecules, which in turn affect cellular processes, like adhesion, migration, proliferation or development (Manou et al., 2019). A balanced and dynamic bidirectional interaction among cells and their surrounding molecules is critical for tissue homeostasis, as well as during wound healing and development. As key components of the ECM, glycosaminoglycans (GAGs) play a key role in tissue hydration, structural scaffolding, as well as cell signaling, hence modulating a wide range of cellular processes (Soares da Costa et al., 2017; Karamanos et al., 2018). Among the GAGs, hyaluronan (HA) is abundant in almost all tissues and outstanding due to its non-sulfated composition and direct cellular secretion into the extracellular space (Dicker et al., 2014; Cowman et al., 2015). Hyaluronan is a linear, negatively charged polysaccharide, build-up of repeating disaccharide units of β-1,4-D-glucuronic acid and β-1,3-N-acetyl-D-glucosamine, reaching a molecular weight between 1 ×10^5^ Da and 1 × 10^7^ Da and a polymer length of up to 25 nm (Toole, 2004). Under physiological conditions, HA chains form a hydrated random coil, occupying a large volume dependent on the bonding between adjacent saccharides and counterbalancing of its negative charge by cations and water (Kutálková et al., 2020).

During physiological or pathological remodeling, lower molecular weight HA fragments accumulate within the tissue by depolymerization via reactive oxygen species and enzymatic cleavage by hyaluronidases (HYAL1-3, PH-20) (Lepperdinger et al., 2001; Wu et al., 2014). Dependent on its molecular weight (MW) and temporal/spatial distribution in the tissue, HA is involved in numerous biological processes, by modulating both its physical properties as well as its direct biochemical signaling (Tavianatou et al., 2019). Hence, HA provides structural support, maintains tissue hydration and serves as a lubricant in certain tissues (Dicker et al., 2014). Furthermore, through interactions with specific HA surface receptors, such as CD44 or RHAMM, HA contributes to the activation of complex cellular signaling pathways involved in cell adhesion, differentiation, proliferation and migration (Itano et al., 2002; Kim and Kumar, 2014; Simpson et al., 2016; Wong et al., 2017). Dysfunction of HA within the ECM is associated with various diseases, such as tumor progression and metastasis, articular degeneration or chronic inflammation (Liang et al., 2016). In the last decades, the role of HA in disease and health was increasingly subject of investigation and led to the development of HA-based biomaterials applied as a dermal filler, drug delivery vehicle or ECM mimetic in tissue engineering (Kim et al., 2017; Hong et al., 2020).

Its high hydrophilicity, biocompatibility, biodegradability and possible extraction from animal independent sources with high purity, qualifies HA to be used as a natural polymer in regenerative medicine (Xu et al., 2012). Particularly advantageous is that HA, as well as all GAGs, exhibits several functional groups that can easily be used for chemical modifications, enabling conjugation of bioactive cues and crosslinking or extending the lifetime by a reduced degradation rate (Köwitsch et al., 2018; Serban and Skardal, 2019). Moreover, a combination of GAGs with a multitude of other materials, whether biologically derived or synthetically manufactured, enables adaption to desired features of biomaterials. Hence, natural and synthetic hydrogels can be applied to either generate ECM mimics or to deconstruct and evaluate how specific ECM parameters (e.g., stiffness, ligand presentation, and dimensionality) provoke particular cellular behaviors.

Despite the increasing knowledge on HA and its bioactivity, the importance of HA’s molecular weight and organization within the ECM is not completely understood. Therefore, it is of particular importance to develop platforms that are suitable to dissect the contribution of HA from additional ECM parameters. Current successful hydrogel systems apply crosslinks between modified HA chains (Burdick and Prestwich, 2011; Broguiere et al., 2016). However, these hydrogel types are unsuitable for studying GAG bioactivities as: (1) the side modifications impair the CD44-HA interactions, important for CD44-dependent cell signaling (Teriete et al., 2004; Banerji et al., 2007; Bencherif et al., 2008; Bhattacharya et al., 2017) and (2) the GAG concentrations govern the network, impeding an independent tuning of the GAG concentrations.

In this context, we designed a hydrogel, which decouples the main hydrogel features from the presence of HA and further GAGs in a user-friendly set-up, which allows analyzing the bioactive role of HA in a defined 3D environment. Careful design of HA-based ECM models could benefit from HA-mediated cellular signaling, which goes beyond exploiting HA as an inert and non-adhesive scaffold. Within our highly controlled model of the ECM, it is possible to dissect and analyze the impact of cell adhesion cues, the abundance of HA and the varying stiffness of soft tissues on cell behavior. Especially because previous research focused mainly on the bioactivity of free diffusing HA in soluble form, the question arises, how a presentation of immobilized HA, as part of an artificial ECM, impacts cellular signaling and behavior.

We applied polyethylene glycol (PEG) hydrogels as a solid base and coupled end-modified GAGs in a controlled manner to the hydrogels. We prepared the hydrogel backbone via thiol-Michael addition reaction between an 8-arm, branched PEG with terminal vinyl sulfone groups and a linear dithiol linker, giving the advantage to work in mild, physiological conditions, suitable to directly embed cells. In general, 8-arm PEG-based hydrogels have a higher shear modulus and swell significantly less compared to those generated from 4-arm PEG (Rezakhani et al., 2020). The synthetic PEG polymer is considered biologically inert and hence prevents unspecific protein adsorption and cell adhesion (Gibas and Janik, 2010). The bioactivity of these hydrogels is modulated by the incorporation of bioactive molecules, like bioactive peptides or growth factors (Ravi et al., 2015). End-thiolated GAGs or cell-adhesive peptides can be conjugated covalently to the backbone, transforming the otherwise inert hydrogel into a highly controlled system, recapitulating features of the ECM environment systematically. This conjugation enables us to investigate the effects of immobilized HA in comparison to freely diffusing HA. Within physiological concentration ranges, the fine-tunable mechanical properties are independent of HA incorporation. Additionally, as the platform is not limited to HA and adaptable for immobilization of other GAGs, it enables the co-presentation of carbohydrates and proteins independently. Within this study, we comprehensively characterized the hydrogel properties and finally demonstrated the versatility of this user-friendly platform for cell experiments with an example: We demonstrate that PEG/GAG hydrogel provides a degradable, 3D cell culture system for analyzing the influence of HA on the sprouting of endothelial cells during angiogenesis.

## Materials and Methods

### Materials for Cell Culture and Hydrogel Preparation

Hyaluronan (sHA, MW 10 kDa) was purchased from LifeCore Biomedical (United States). Dermatan sulfate (DS, MW 30 kDa) was purchased from Merck (Germany). Chondroitin sulfate A (CS, MW 10–30 kDa) and heparin (Hep, MW 16.5 kDa) were obtained from Biosynth-Carbosynth (United Kingdom). PEG-dithiol (MW 1 kDa), methylcellulose (4,000 cP) and OptiPrep (density gradient medium) were purchased from Sigma-Aldrich (Germany). The linRGD peptide (sequence: GCGWGRGDSPG) was purchased from PSL GmbH (Germany), the crosslinker peptide (sequence: GCREGPQGIWGQERCG) from Pepscan (Netherlands). 8-arm PEG with terminal vinyl sulfone groups (PEG-VS, MW 20 kDa, tripentaerythritol core) was obtained from JenKem Technology (United States). Recombinant human VEGF was purchased from R&D Systems (United States). Human umbilical vein endothelial cells (HUVECs, pooled donor, cryopreserved), endothelial cell growth medium (ECGM) and basal medium were purchased from Promocell (Germany).

### Glycosaminoglycan Modifications

sHA was functionalized at the reducing N-acetylglucosamine unit according to the procedure of Lee et al. (2008). Briefly, sHA (100 mg), cysteamine hydrochloride (120 mg, 100 mM, Sigma-Aldrich, Germany) and sodium chloride (467.5 mg, 400 mM, Merck, Germany) were dissolved in borate buffer (20 mL, 100 mM, pH 8.5, Sigma-Aldrich, Germany) for 2 h at room temperature. After adding sodium cyanoborohydride (251 mg, 200 mM, Sigma-Aldrich, Germany), the reaction mixture was stirred for 5 days at 40°C in an oil bath. Afterward, the reaction mixture was incubated with dithiothreitol (DTT, 150 mg, Sigma-Aldrich, Germany) for 2 h at 40°C. The reaction mixture was dialyzed (MWCO: 3500 Da, Carl Roth, Germany) against acidified deionized water with sodium chloride (pH 5) for 2 h and then against deionized water for 2 days. The thiolated sHA (sHA-eSH) was recovered by freeze-drying and stored at −80°C. For verification, the end-terminal thiol modification of sHA-eSH was quantified to 81.4 ± 3.0% by an adapted colorimetric Ellman’s assay (Ellman, 1958). The same procedure was applied for thiolation of the polysaccharides chondroitin sulfate A (CS), dermatan sulfate (DS) and heparin (Hep), however, without sodium chloride in the reaction mix and dialysis with deionized water only. Terminal thiolation was quantified to 73.6% ± 0.4% (CS), 108.6% ± 9.3% (DS), 108.8% ± 2.1% (Hep) by the colorimetric Ellman’s assay.

For the fluoresceinamine (FA, isomer 1, Sigma-Aldrich, Germany) fluorophore conjugation, sHA (50 mg) and 1-ethyl-3-[3-dimethylaminopropyl] carbodiimide (EDC, 10 mg, Sigma-Aldrich, Germany) were dissolved in PBS (15 mL, 100 mM, pH 5.8) for 30 min and FA (2.5 mg/mL in DMSO) was added dropwise (final conc. 0.1 mg/mL). The reaction mixture was incubated at room temperature for 5 h and dialyzed as before. To synthesize an end-thiolated and labeled sHA-eSH, the same labeling protocol was performed before the thiolation reaction. To determine the success of this reaction, the degree of labeling (DOL) was determined. Therefore, the fluorescence intensity of the products and a serial dilution of the fluorophore was measured at 488 nm/533 nm in the plate reader (Spark, Tecan, Swiss). The degree of labeling is specified as molar percentage of dye to HA monomer content (sHA: 14.9% ± 1.0%, sHA-eSH: 0.07% ± 0.02%).

### Hydrogel Formation

Preparation of hydrogels was accomplished through thiol-Michael addition reaction of an 8-arm PEG with terminal vinyl sulfone groups (PEG-VS) and a PEG-dithiol crosslinker. Hydrogels were formed with a series of PEG polymer concentrations (3.5 w/v%, 5.5 w/v%, and 7.5w/v%). For example to prepare 100 μL of 3.5 w/v% non-degradable hydrogels, 14.6 μL of a 20 w/v% (10 mM) PEG-VS stock was mixed with 29.2 μL of a 2 w/v% (20 mM) crosslinker stock in 56.2 μL PBS. For the preparation of functionalized hydrogels, PEG-VS was incubated together with the adhesive RGD motif (0.5 mM, linRGD) and end-thiolated GAGs (0–1 mg/ml) for 20 min in PBS. This functionalization step will result mainly in single functionalized PEG-VS, since the added thiol containing species are far less than equivalence [for highest functionalization with 500 μM linRGD and 1 mg/mL sHA-eSH (76.9 μM) the molar VS:SH ratios are 1:0.049 (3.5 w/v%), 1:0.031 (5.5 w/v%) and 1: 0.023 (7.5 w/v%)]. In a polymerization step, the preincubated PEG-VS solution was mixed with crosslinker and incubated for 1 h at 37 °C. Thereby, the molar ratio of reactive thiol groups (total thiols from crosslinker and functionalization) and vinyl sulfone groups (from PEG-VS) were stoichiometrically balanced (see detailed calculation in Supplementary Material). Similar degradable hydrogels were crosslinked with a peptide, which is degradable by a range of matrix metalloproteinases (MMP-cleavable peptide). Semi-degradable hydrogels were prepared with a 1:1 molar ratio of the PEG-dithiol crosslinker and the MMP-cleavable peptide. The crosslinker concentrations for non-degradable hydrogels are summarized in Table 1 (for other crosslinkers see Table 1 in Supplementary Material). The stock solutions of sHA-eSH and the MMP-cleavable peptide, dissolved in PBS, were adjusted to pH 7.4 with 1 M NaOH.

**TABLE 1:** Crosslinker concentrations (in mM) for non-degradable hydrogels.

Crosslinker	Functionalization	3.5 w/v%	5.5 w/v%	7.5 w/v%
Non-degradable	PEG only	11.7	18.3	25.0
	500 μM linRGD	11.3	17.9	24.6
	500 μM linRGD + 76.9 μM sHA-eSH	11.2	17.9	24.5

### Time Resolved Conjugation of End-Thiolated Hyaluronan and linRGD to Polyethylene Glycol-Vinyl Sulfone

The Measure-IT^TM^ Thiol Assay Kit (Invitrogen, M30550, Thermo Fischer Scientific, Germany) was used to monitor the conjugation of PEG-VS with either sHA-eSH or linRGD by detecting a decrease in thiol groups over time. The kit was used according to the manufacturer’s instructions. sHA-eSH or linRGD peptide was reacted with PEG-VS for up to 1 h, before reacting with 100 μL working solution of the assay. The decrease in thiol groups is presented as a percentage of initial thiols over time. Concentrations are according to a functionalization step of a 5.5 w/v% hydrogel with 0.5 mM linRGD and 1 mg/mL sHA-eSH.

### Glycosaminoglycan Detection in Hydrogels

The physical entrapment of sHA within a pure PEG hydrogel was assessed. Therefore, 3.5 w/v% hydrogels (10 μL) were prepared with different amounts of either a soluble fluorescently-labeled sHA or an end-thiolated fluorescently-labeled sHA (0–1 mg/mL). The fluorescence intensity of the hydrogels after polymerization was measured in the plate reader (488 nm/533 nm). After washing in 70 μL PBS at 37°C for 24 h in the dark (PBS was exchanged after 5 h and 20 h), the fluorescence intensity of the hydrogels was measured again in the plate reader using the same settings. For comparison, fluorescence intensities of hydrogels before and after washing were plotted for both soluble sHA and immobilized sHA-eSH.

Immobilized GAGs within hydrogels were also visualized by staining with the cationic carbocyanine dye Stains-All (Sigma-Aldrich, Germany). 3.5 w/v% hydrogels (10 μL) were prepared with the PEG-dithiol linker and functionalized with 1 mg/mL end-thiolated GAGs in triplicates. Hydrogels were incubated for 24 h in 50 μL TRIS-borate-EDTA buffer (89 mM tris(hydroxymethyl)aminomethane base (TRIS), 89 mM boric acid and 2 mM ethylenediaminetetraacetic acid (EDTA), pH 8.3, all Sigma-Aldrich, Germany) with two buffer exchanges. Hydrogels were stained with 50 μL of a Stains-All solution (0.005 w/v% in 70 v/v% ethanol) for 4 h and destained for 2 × 1 h with 50 μL 10 v/v% ethanol, before taking an image.

Carbazole-based quantification of hyaluronic acid within hydrogels comprised hydrolyzation of sHA in its monomers and a color reaction of glucuronic acid with carbazole (Plätzer et al., 1999; Frazier et al., 2008). A serial dilution of sHA in water in triplicates was used as an external standard. Lyophilized PEG hydrogels (90 μL) functionalized with 0.5 mM linRGD and varying amounts of sHA-eSH as well as 50 μL of serial dilution samples were incubated with 200 μL 25 mM sodium tetraborate in sulfuric acid at 100 °C for 10 min and cooled at room temperature for 15 min. Then 50 μL of a 0.125% carbazole solution (Carl Roth, Germany) in absolute ethanol was added. After incubation at 100°C for 10 min and at room temperature for 15 min, the absorbance was measured at 550 nm in a plate reader.

### Crosslinking Efficiency and Quantification of Thiols in Hydrogels

To quantify the crosslinking efficiency, all types of hydrogels were prepared in triplicates, freeze-dried and weighed [m(non-extracted)]. Then hydrogels were incubated in PBS for 2 days at 37°C, freeze-dried and weighed again (m(extracted)). The crosslinking efficiency was calculated by dividing m(extracted) by m(non-extracted).

The percentage of free thiols within hydrogels (40 μL) was determined using Ellman’s assay. Hydrogels and an unpolymerized sample (only thiol linker in buffer, to determine the initial thiol concentration) were prepared in triplicates. TRIS buffer (784 μL, 1 M, pH 8) and Ellman’s reagent (784 μL, 2 mM 5,5′-Dithiobis(2-Nitrobenzoic-acid) and 50 mM sodium acetate in water, both Sigma-Aldrich, Germany) was added per sample. After incubation for 20 min at 350 rpm, the absorption of the supernatant was measured at 412 nm. Values are in percentage of free thiol groups measured in the unpolymerized sample.

### Equilibrium Swelling and Mesh Size Calculations

After measuring the mass of hydrogels (90 μL) swollen in PBS or endothelial cell growth medium, hydrogels were freeze-dried and weighed again. The swelling ratio (Q_*m*_) was calculated as the ratio of the swollen hydrogel mass by its dry mass. Their mesh size ξ was calculated based on the Flory-Rehner theory, as described previously (Peppas et al., 2000; Zustiak and Leach, 2010). First, the molecular weight between crosslinks ($\bar{M_{c}}$) was calculated by:


$$\frac{1}{\bar{{\text{M}}_{c}}}=\frac{2}{\bar{{\text{M}}_{n}}}-\frac{\frac{\bar{v}}{V_{1}}\left(\text{ln}\left(1-{\text{v}}_{2,s}\right)+{\text{v}}_{2,s}+{\text{X}}_{1}{\text{v}}_{2,s}^{2}\right)}{{\text{v}}_{2,r}\left[{\left(\frac{v_{2,s}}{v_{2,r}}\right)}^{1/3}-\frac{v_{2,s}}{2v_{2,r}}\right]}$$

Where $\bar{M_{n}}$ is the number-average molecular weight of the non-crosslinked polymer chains, *V*_1_ is the molar volume of the solvent (18 cm^3^/mol for water), $\bar{v}$ is the specific volume of the polymer (ρ_*s*_/ρ_*p*_ with polymer density ρ_*p*_ = 1.12 g/cm^3^ and solvent density ρ_*s*_ = 1.0 g/cm^3^), *v*_2,*s*_ is the polymer volume fraction in swollen hydrogels, *v*_2,r_ is the polymer volume fraction in the relaxed state (immediately after polymerization), *X*_1_ is the polymer-solvent interaction parameter (0.426 for PEG in water) (Lu and Anseth, 2000). Then, the mesh size was calculated by:


$$\xi =\frac{1}{\sqrt[{3}]{{\text{V}}_{2,s}}}l\sqrt{\frac{2{\text{C}}_{n}\bar{{\text{M}}_{c}}}{{\text{M}}_{r}}}$$

Where *l* is the average bond length across the backbone of the polymer (0.146 nm for PEG), *C*_*n*_ is the Flory characteristic ratio (4 for PEG polymers) and *M*_*r*_ is the molecular weight of the repeating polymer units (44 g/mol for PEG) (Mellott et al., 2001). Thereby, contributions of the peptide crosslinker to the Flory characteristic ratio and the molecular weight of the repeating units were neglected according to the approach of Schwartz et al. (2009) and Rehmann et al. (2017), who studied similar hydrogel systems.

### Rheological Measurements of Swollen Hydrogels

Material properties were assessed using a rotational rheometer (Kinexus Pro+, Malvern, United Kingdom) equipped with an Active Hood Peltier plate cartridge and a parallel-plate geometry (8 mm) with a solvent trap. For all hydrogel conditions, a strain sweep between 0.1 and 100% at a constant frequency of 1 Hz and a frequency sweep with frequencies between 0.01 and 100 Hz at a constant shear strain was performed to determine the linear viscoelastic regime. Swollen hydrogels were fixed between the plates by a normal force set to 0.1 N (3.5 w/v%) or 0.2 N (5.5 w/v% and 7.5 w/v%). All stiffness measurements were performed at 25°C, for 2 min with an interval of 5 s with a frequency of 1 Hz and a shear strain of 5%. The Young’s modulus *E* is related to the shear modulus *G* by:


E=G 2(1+v)

with a Poisson’s ratio *v* assumed as 0.5. The gelation time was determined for several hydrogel conditions. The evolution of the storage and loss modulus (G′ and G″, respectively) was recorded at 37°C with an interval time of 2 min over a period of 1 h, at a frequency of 1 Hz and a constant strain of 1%.

### Enzymatic Degradation of Hydrogels

3.5 w/v% hydrogels (40 μL) were prepared as described and swollen in HEPES buffered saline (10 mM 2-[4-(2-hydroxyethyl)piperazin-1-yl]ethanesulfonic acid (HEPES, Sigma-Aldrich, Germany), 137 mM sodium chloride, 1 mM calcium dichloride (Carl Roth, Germany), 3 mM sodium azide (Riedel-de Haën, Germany), pH 7.4) overnight. Ca^2+^ ions are necessary for collagenase activity. Hydrogels were then incubated with 300 μL buffer or 2 mg/mL collagenase (Type 1, from clostridium histolyticum, Sigma-Aldrich, Germany) at 37°C and their mass was weighed over 48 h. After each measurement, solutions were renewed.

### Quartz Crystal Microbalance With Dissipation Monitoring

Experiments were performed with the manually handled Q-Sense E4 instrument, equipped with a 4-channel system, open modules and gold-coated quartz crystal electrodes (AT cut, 4.95 MHz, Biolin Scientific AB, Sweden). Electrodes were cleaned in a washing solution consisting of 5:1:1 (volume parts) deionized water, 25% ammonia (Merck, Germany) and 30% hydrogen peroxide (Sigma-Aldrich, Germany) at 75°C for 10 min, followed by UV/ozone treatment (ProCleaner, Bioforce Nanosciences, IA, United States) for 10 min. The frequency and the dissipation of the 3rd, 5th, 7th, 9th, 11th, and 13th overtones were monitored. After setting a baseline in PBS, 200 μL of the desired solution was pipetted on the electrodes. The solutions remained on the electrodes until a stable plateau in both the dissipation and frequency was maintained for at least 5 min. Each adsorption step was followed by washing the electrodes with PBS to remove non-interacting molecules. Each sample was tested in duplicates. For validating CD44-sHA interaction, protein G (BioVision, CA, United States), sHA/sHA-eSH, CD44-Fc-tag (Acro Biosystems, DE, United States) and bovine serum albumin (Sigma-Aldrich, Germany) concentrations were 10 μg/mL, 0.5 mg/mL, 3 μg/mL, and 0.1 mg/mL in PBS, respectively. Data of the 7th overtone are shown.

### Cell Culture and Generation of Endothelial Cell Spheroids

Human umbilical vein endothelial cells (HUVECs) were cultured in endothelial cell growth medium (ECGM) at 37°C with humidified 5% CO_2_ atmosphere up to passage 6. After reaching 90% confluency HUVECs were detached with 0.05% trypsin-EDTA (Thermo Fisher Scientific, Germany), collected, centrifuged at 230 × g for 3 min and reseeded at an appropriate density. Endothelial cell spheroids were generated using the hanging-drop method. HUVECs were harvested and suspended in ECGM containing 0.25 w/v% methylcellulose. After deposition of 25 μL drops on the lid of a humidified chamber, the hanging droplets were cultivated for 24 h. Under these conditions, the suspended cells aggregate and form a single spheroid of defined size and cell number (600 cells per spheroid).

### *In vitro* Angiogenesis Assay

For the *in vitro* angiogenesis assay, spheroids were harvested and embedded into degradable 3.5 w/v% PEG hydrogels, functionalized with 0.5 mM linRGD and varying amounts of sHA-eSH (0–1 mg/mL). Hydrogels were prepared as described previously with 15 v/v% OptiPrep to prevent sedimentation of spheroids prior to polymerization of the hydrogel. For the polymerization step, the spheroid and crosslinker solutions were added to the functionalized PEG-VS and 10 μL hydrogels were formed in 96-well angiogenesis μ-plates (Ibidi GmbH, Germany). After polymerization, hydrogels were overlaid with 70 μL cell culture medium (basal medium with 15% FBS). For stimulation of the HUVECs, the cell culture medium was supplemented with 50 ng/mL VEGF_165_. The gels were incubated at 37°C in a humidified 5% CO_2_ atmosphere and imaged after 48 h. Phase-contrast images were acquired with a Zeiss Primovert (Zeiss, Germany), equipped with an Axiocam 208 color and 20 × objective (Zeiss, Germany, Plan-Achromat, Ph1, 20 ×/0.30). Sprouting was quantified by measuring the cumulative sprout length, which had grown out of each spheroid, using the imaging software FIJI.

### Statistical Analysis

Data are presented as mean ± standard deviation. One-way analysis of variance (ANOVA) followed by Tukey *post-hoc* test was used to compare between conditions with GraphPad Prism 8.0 software.

## Results

### Presentation of Glycosaminoglycans Within a Highly Controllable and Tunable Set-Up

#### Conjugation of Glycosaminoglycans to Polyethylene Glycol Hydrogels

**FIGURE 1:**
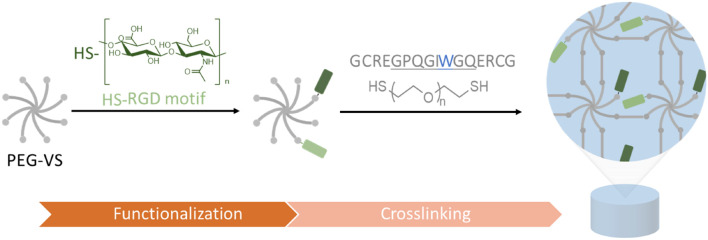
Schematic illustration of PEG/GAG hydrogel formation. Branched 8-arm poly (ethylene glycol) PEG with terminal vinyl sulfone groups (VS) is functionalized with end-thiolated GAGs and peptide binding motifs (e.g., RGD) via thiol-Michael addition reaction. Following this functionalization, PEG macromolecules are crosslinked with a PEG-dithiol linker or matrix-metalloprotease (MMP)-cleavable di-cysteine peptide. The peptide linker sequence is derived from collagen with a single amino acid exchange from A to W (blue) for a higher enzyme affinity and enhanced degradability (cleavage site underlined).

Within this study, we developed and characterized a PEG/GAG hydrogel, which helps us to investigate the bioactive role of HA and further GAGs as part of a well-defined 3D environment. By altering independently the matrix stiffness as well as the GAG and peptide presentation in a modular set-up, we can better understand the interplay of these parameters and their impact on cellular behaviors. For this purpose, PEG hydrogels were formed as a solid base and end-thiolated GAGs were conjugated in a controlled manner to the hydrogel. PEG hydrogels were prepared via thiol-Michael addition reaction of an 8-arm branched PEG-vinyl sulfone (PEG-VS) and a linear dithiol linker, as described in previous studies (Lutolf et al., 2003a). Synthesis of the PEG hydrogels is conducted in two steps (Figure 1). In a first functionalization step, bioactive ligands are conjugated to a branched 8-arm PEG monomacromer. In a subsequent gelation step, a dithiol linker molecule is added and the gel is polymerized.

**FIGURE 2:**
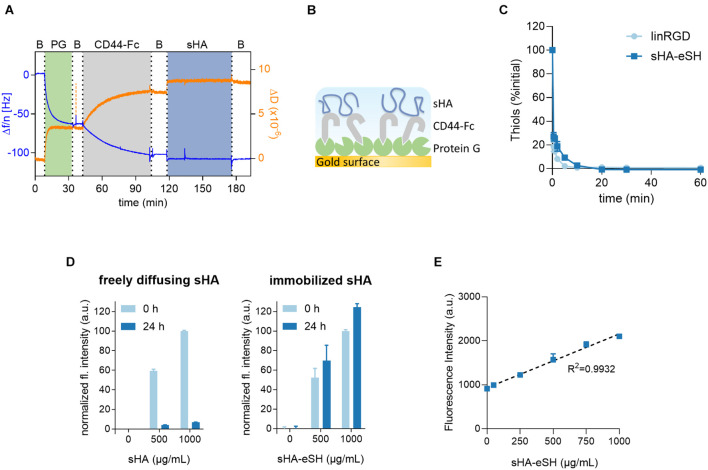
**(A)** QCM-D measurement confirms CD44-sHA interaction. The binding experiment shows the formation of a stable adlayer of protein G (PG) on a gold surface, followed by immobilization of Fc-tagged CD44 and its binding to sHA. Data from the 7th overtone are shown, frequency changes depicted in blue and dissipation changes in orange. **(B)** Schematic diagram of immobilization strategy on QCM-D sensor. **(C)** Kinetics of thiol-Michael addition reaction of sHA-eSH and linRGD. Progression of the conjugation to 8-arm PEG-VS macromonomers was monitored over time by detecting the decrease of free terminal thiol groups; mean and standard deviation of triplicates are presented. **(D)** Detection of varying amounts of fluorescently-labeled hyaluronan within hydrogels after incubation in PBS for 24 h. 3.5 wt/vol% PEG hydrogels with varying amounts of fluorescent sHA (soluble sHA) or sHA-eSH (immobilized sHA) were prepared by thiol-Michael addition reaction. **(E)** Fluorescence intensity values of hydrogels after washing in PBS are plotted over the hyaluronan concentration per mL of hydrogel solution; mean and standard deviation of quintuplicates are presented.

Prior to PEG/HA hydrogel formation, the interaction of the small hyaluronan oligosaccharides (sHA), used within this study, and its standard transmembrane receptor CD44 was proven in a label-free QCM-D experiment (Figures 2A,B). Dissipation and frequency changes monitored over time indicate binding of sHA to the receptor CD44, immobilized via a recombinant Fc-tag to a protein G adlayer. Corresponding dissipation and frequency shifts were observed with sHA-eSH (Supplementary Figure 1). After verifying the sHA-CD44 interaction, the presentation of sHA within a 3D scaffold was achieved with a straightforward method: sHA was modified at the reducing end with a short thiol linker (named sHA-eSH or end-thiolated sHA) and subsequently immobilized to PEG-VS, a building block for the hydrogel formation. After successful end-thiolation of sHA, its conjugation to the branched 8-arm PEG-VS was tested. The decrease in terminal free thiol groups of the sHA-eSH, due to its conjugation to PEG-VS, was detected in a time resolved assay (Figure 2C). An exponential decay of terminal thiols proved the conjugation of end-thiolated sHA to PEG-VS. A comparable reaction rate was detected for the conjugation of the cell adhesive linear RGD (linRGD) peptide motif (Figure 2C). A complete conversion of both biomolecules is achieved within 20 min. Consequently, the functionalization was left to proceed for 20 min before addition of the crosslinker. In a subsequent step, these functionalized PEG macromolecules are crosslinked into a network by addition of a dithiol linker.

Due to sHA’s flexibility and small size, its physical entrapment within PEG hydrogels was assessed. Therefore, 3.5 w/v% PEG hydrogels were loaded with varying amounts of soluble fluorescently-labeled sHA. The fluorescence intensities of these hydrogels were measured before and after washing in PBS. A drop in the detected fluorescence intensities of these hydrogels was observable when hydrogels were incubated in PBS for 24 h (Figure 2C). This indicates that sHA freely diffuses out of the hydrogel. Next, hydrogels were prepared with a fluorophore-labeled and end-thiolated sHA. In this case, similar fluorescence intensities of the hydrogels before and after incubation in PBS were observed (Figure 2D). It can be concluded that thiol-mediated immobilization of sHA-eSH to the PEG backbone prevents the diffusion of sHA out of the hydrogel. These two experiments in combination demonstrate the necessity for conjugating sHA to the PEG hydrogel for a reliable presentation of HA as part of a 3D microenvironment. Moreover, a very good correlation between an increasing amount of sHA-eSH within the hydrogel and the detected fluorescence intensity values was observable (Figure 2E).

**FIGURE 3:**
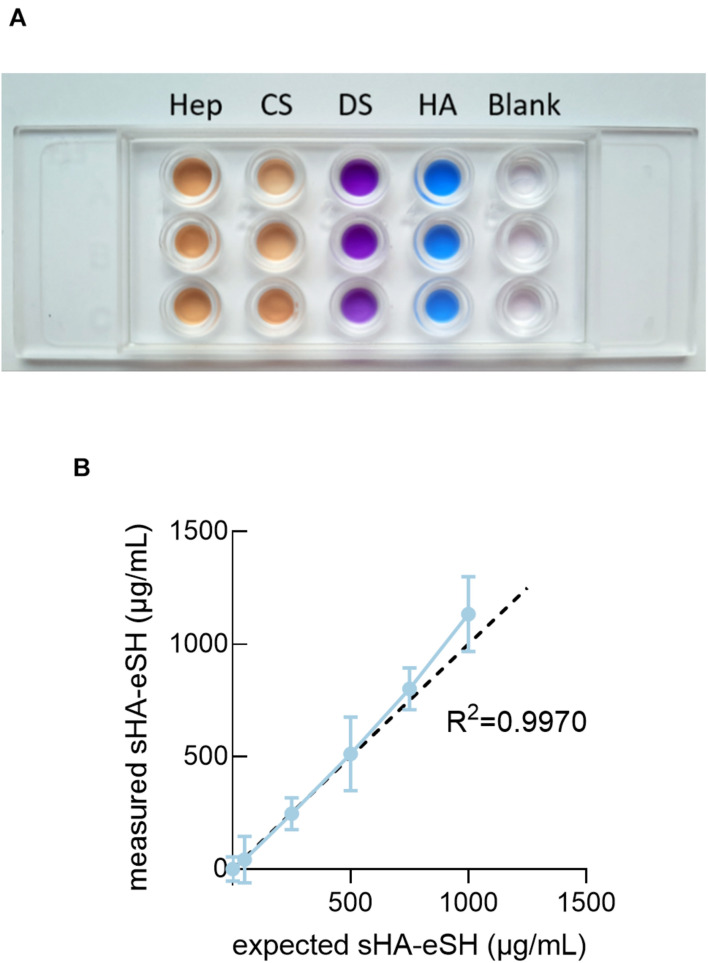
Detection of GAGs in PEG hydrogels. **(A)** PEG hydrogels were prepared with 1 mg/mL end-thiolated GAGs [heparin (Hep), chondroitin sulfate (CS), dermatan sulfate (DS) and hyaluronan (HA)] and stained with Stains-All after incubation in borate buffer for 24 h. Stained gels reveal a detection of GAGs with distinct colors. Blank samples were prepared with water and appeared colorless. **(B)** Quantification of sHA-eSH in a carbazole-based assay. PEG hydrogels were functionalized with 0.5 mM linRGD and varying amounts of sHA-eSH (0–1 mg/mL). Measured HA-eSH concentrations are plotted against expected concentrations. Hydrogels were swollen in PBS > 20 h and lyophilized, before performing the assay; mean and standard deviation of triplicates/quadruplicates are presented.

Additionally, this approach of immobilizing end-thiolated sHA is adaptable for immobilization of other end-thiolated GAGs. To demonstrate this, further common GAGs, more precisely chondroitin sulfate, dermatan sulfate and heparin, were modified with an end-terminal thiol group and their presence within hydrogels was detected by staining with the cationic carbocyanine dye Stains-All. Stains-All is a metachromatic dye capable of staining different GAG types in distinct and characteristic colors (Andrade et al., 2018). Hydrogels were prepared with 1 mg/mL of end-thiolated GAGs, incubated for 24 h in borate buffer and stained with Stains-All. Stains-All incubation showed distinct colors for each of the GAG in comparison to the almost transparent water control (Figure 3A). It can be concluded, that the approach of immobilizing sHA via a short thiol linker is extendable to further GAGs. Next to these qualitative verifications, immobilized sHA-eSH within hydrogels was quantified in absolute numbers by a colorimetric assay. Measured sHA-eSH concentrations of each sample are plotted against the expected theoretical values (Figure 3B). Overall, a good correlation occurs between these values. In accordance with the previous diffusion experiments, a presentation of hyaluronan as part of a network is achievable. Finally, this immobilization strategy enables the independent co-presentation of GAGs and peptides. This facilitates the direct sensing of biological signals by the cells within the otherwise inert PEG hydrogels.

#### Physicochemical Characterization of Hydrogels With Varying Amounts of End-Thiolated Hyaluronan

The physical and chemical properties of PEG/GAG hydrogels were analyzed. Detailed characterization referred to small hyaluronan oligosaccharides (sHA), the most studied GAG type. Non-functionalized hydrogels were named “PEG only”, while hydrogels classified as “0–1,000 μg/mL sHA-eSH” are co-functionalized hydrogels with a constant adhesive linRGD motif concentration (0.5 mM) and varying amounts of hyaluronic acid.

**FIGURE 4:**
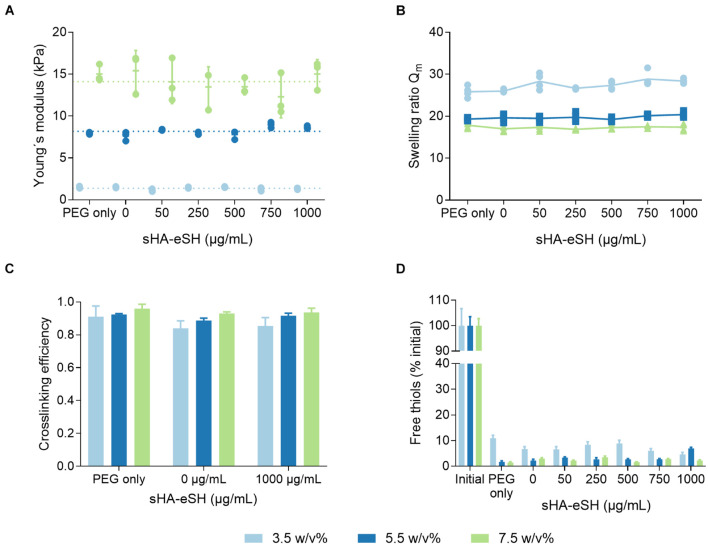
Physicochemical characterization of PEG/HA hydrogels. Hydrogels were prepared in three different PEG polymer concentrations by thiol-Michael addition reaction with a PEG-dithiol crosslinker. Hydrogels were unmodified (PEG only) or functionalized with 0.5 mM linRGD and varying concentrations of sHA-eSH (0–1 mg/mL). **(A)** Bulk elasticity. The Young’s moduli of hydrogels were determined by shear rheological measurements and range from 1.4 to 14.1 kPa, dependent on the PEG polymer concentration; mean and standard deviation of triplicates are presented. **(B)** Swelling ratios. Hydrogels were equilibrium-swollen in PBS; mean and standard deviation of quadruplicates are presented. **(C)** Crosslinking efficiency based on the sol-gel fraction of PEG/HA hydrogels; mean and standard deviation of triplicates are presented. **(D)** Detection of free thiol groups within non-degradable hydrogels using Ellman’s reagent. Initial corresponds to an unpolymerized hydrogel (all thiol groups free); mean and standard deviation of triplicates are presented.

Mechanical properties of hydrogels were tuned by varying the PEG polymer concentration (3.5 w/v%, 5.5 w/v%, and 7.5 w/v%), modulating the number of backbone polymers in a given volume within the hydrogel network. The Young’s moduli of the PEG/HA hydrogels generally increase with an increase in the total polymer fraction, as determined by rheological measurements (Figure 4A). It was revealed that the incorporation of both types of bioactive molecules, linRGD motif (0.5 mM) and sHA-eSH (up to 1 mg/mL, equals 0.077 mM), barely influences the Young’s modulus. Hydrogels have Young’s moduli of 1.4 kPa ± 0.2 kPa (3.5 w/v%), 8.2 kPa ± 0.5 kPa (5.5 w/v%), and 14.1 kPa ± 2 kPa (7.5 w/v%), and thus are within the range of physiological elasticity of soft tissues (Engler et al., 2006). The process of polymerization was monitored over time in rheological measurements (Supplementary Figure 2, Supporting Information). The gel point, defined as the time of crossover of storage modulus G′ and loss modulus G″, was below 2 min under the present conditions (pH 7.4, 37°C). Both storage and loss modulus increase until reaching a plateau, indicating complete gelation. The gelation time depends mainly on the total polymer fraction, and less on the applied degree of functionalization [15 min (7.5 w/v%), 20 min (5.5 w/v%), and 25 min (3.5 w/v%)]. Due to an equal stoichiometric ratio of free VS to SH groups, the PEG-VS and crosslinker concentrations increase with an increase of the PEG polymer concentration from 3.5 w/v% to 7.5 w/v%. Additionally, with higher degree of functionalization the crosslinker concentration is slightly reduced (Table 1). Overall, applied gelation times of 60 min were sufficient for exhaustive crosslinking.

As part of a physicochemical characterization, the swelling behavior was investigated including the swelling ratio Q_*m*_ in PBS and cell culture medium. Swelling ratios for equilibrium-swollen hydrogels in PBS were in general higher in comparison to hydrogels swollen in cell culture medium (Table 2). In both cases, the swelling ratio decreased with an increase in hydrogel stiffness and was independent of a modification with linRGD and sHA-eSH (Figure 4B). A further parameter to characterize the network structure is the mesh size, defined as the linear distance between neighboring crosslinks. Calculations of the mesh size are based on swelling ratios and follow the trend observed for the bulk elasticity as well as for swelling ratios. With rising PEG polymer concentrations, the hydrogel stiffness increases, while the mesh size decreases. Thereby, swelling ratios, as well as mesh sizes for functionalized hydrogels are unaffected by the varying amounts of sHA-eSH and the presence of the adhesive linRGD motif (Supplementary Figure 2). Thus, the mean and standard deviation are shown, independent of the functionalization (Table 2). In each case, the mesh size is larger than the bulk size of the immobilized hyaluronan with ∼4.4 nm (Takahashi et al., 1999).

**TABLE 2:** Elasticity, swelling ratio and mesh size for PEG/HA hydrogels.

w/v%	3.5	5.5	7.5
Elasticity [kPa]	1.4 ± 0.2	8.2 ± 0.5	14.1 ± 2.0
Q_*m*_ (PBS)	27.3 ± 1.5	19.7 ± 0.7	17.3 ± 0.6
Q_*m*_ (medium)	22.0 ± 0.6	17.5 ± 1.0	15.8 ± 2.6
Mesh size [nm]	11.7 ± 0.6	9.1 ± 0.4	8.2 ± 0.3
Gelation [min]	25	20	15

Defects in the network structure occur either based on non-reacted groups or the formation of disulfide bonds, loops and entanglements. To get more insight into the network structure, the sol fraction and crosslinking efficiency were derived by weight changes due to the extraction of non-reacted PEG. The crosslinking efficiency was found to correlate with the PEG polymer concentration of the hydrogel formulation (Figure 4C). This is in accordance with the trends for the bulk stiffness, swelling ratios and mesh size. Overall, hydrogels show high reaction conversion rates. Crosslinking efficiency was lowest for 3.5 w/v% hydrogels with 0.87 ± 0.05 and highest for 7.5 w/v% hydrogels with 0.94 ± 0.02. The crosslinking efficiency seems to be unaffected by the modifications with linRGD and sHA-eSH. Additionally, to get more insights into the network defects, the number of free thiol groups was assessed immediately after polymerization. Free thiols were quantified in an Ellman’s assay, and correspond to non-reacted crosslinker molecules within a hydrogel (Figure 4D). Hydrogels prepared with increasing PEG polymer concentrations showed lower amounts of free thiols, following the trend observed for the crosslinking efficiency before. Again, the reaction conversion seems unaffected by the functionalization with linRGD and sHA-eSH.

#### Matrix-Metalloprotease-Cleavable Peptide Linker: Degradable Hydrogels for 3D Cell Culture

To recapitulate the ECM degradability, essential for several biological processes, PEG-VS macromeres are often crosslinked with an MMP-cleavable di-cysteine peptide. The MMP-sensitive peptide (16 amino acids) is derived from the cleavage site of natural collagen with a single amino acid mismatch for enhanced degradation (Lutolf et al., 2003b; Patterson and Hubbell, 2010). The so far applied PEG-dithiol linker of 1 kDa with a contour length of ∼6.16 nm (Oesterhelt et al., 1999) was chosen according to the contour length of the peptide (∼6.08 nm; Ainavarapu et al., 2007). Similar contour lengths were preferred to achieve similar network structures for both types of crosslinker.

**FIGURE 5:**
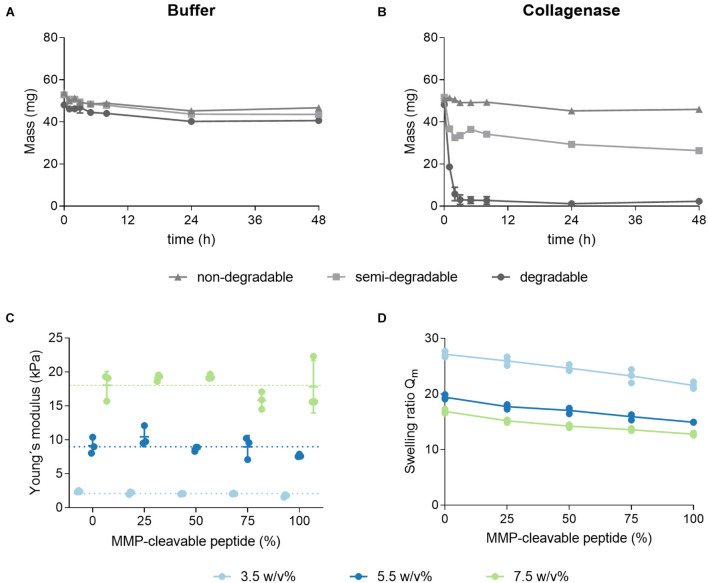
Effect of a stepwise replacement of the PEG-dithiol linker with the MMP-cleavable peptide on degradation, elasticity and swelling ratio. **(A,B)** Enzymatic and hydrolytic degradation of non-degradable, semi-degradable and degradable hydrogels. 3.5 w/v% hydrogels were prepared by thiol-Michael addition reaction using either the PEG-dithiol (non-degradable), a mixture of PEG-dithiol and the MMP-cleavable di-cysteine peptide (semi-degradable, molar ratio 1:1) or the peptide alone (degradable). Hydrogels were swollen overnight in buffer and mass changes due to enzymatic degradation were monitored over 48 h when incubated in buffer **(A)** or with collagenase **(B)**. **(C,D)** Hydrogels were formed with three different PEG polymer concentrations by thiol-Michael addition reaction with a stepwise replacement of the PEG-dithiol crosslinker by the MMP-cleavable di-cysteine peptide. **(C)** The Young’s moduli of hydrogels were determined by shear rheological measurements. **(D)** Swelling ratios of hydrogels with increasing degradability. Swelling ratios were calculated by dividing the mass of the equilibrium-swollen hydrogel by the dry mass; mean and standard deviation of triplicates are presented.

Degradation characteristics of hydrogels, crosslinked with three different ratios of the MMP-cleavable di-cysteine peptide and the PEG-dithiol linker, were evaluated (Figures 5A,B). Previously swollen hydrogels were enzymatically degraded with collagenase, while their change in total weight was monitored over time. As a control, hydrogels were incubated in buffer over the same period. Hydrogels incubated in buffer were almost constant in weight, thus indicating the stability of the hydrogels over 48 h, regardless of the crosslinker composition. Hydrogels formed with the MMP-cleavable peptide linker (degradable) show complete enzymatic degradation within 2 h. Hydrogels, crosslinked with a linker mix of the PEG-dithiol and MMP-cleavable peptide (semi-degradable, molar ratio 1:1), lose their mass to a certain degree upon incubation with collagenase. This mass decrease might be explained by the partial degradation of the network. PEG-dithiol crosslinked hydrogels maintain their weight during incubation with collagenase, verifying an expected non-degradability. Altogether, the degradability of the hydrogels can be controlled by the ratio of degradable peptide linker to non-degradable PEG-dithiol linker. Next, the influence of a stepwise replacement of PEG-dithiol with the MMP-cleavable di-cysteine peptide on the physicochemical characteristics including the mechanical properties and swelling behavior was investigated.

As seen previously, Young’s moduli in shear rheological measurements of hydrogels, prepared with varying ratios of the PEG crosslinker and the MMP-cleavable di-cysteine peptide, highly depend on the PEG polymer concentration. The impact of the crosslinker ratio on the Young’s moduli seems limited (Figure 5C). The observed swelling ratios for these hydrogels decrease with an increasing percentage of the peptide linker (Figure 5D). This trend is more pronounced for the softer hydrogels (3.5 w/v%).

Overall, the developed PEG/GAG hydrogel system allows a presentation of GAGs in physiological concentrations independent from the presentation of peptides as cell adhesive binding motifs. Moreover, the hydrogels are fine-tunable in their degradability via the MMP-sensitivity of the dithiol linker and their stiffness via the PEG polymer concentration. Including GAGs as part of the natural ECM in synthetic hydrogels enables either the generation of ECM mimics or the deconstruction and evaluation of how specific ECM parameters induce specific cellular behaviors with a bottom-up approach.

### Application as Well-Defined Artificial 3D Microenvironment

#### Polyethylene Glycol/Hyaluronan Hydrogels Support Endothelial Cell Sprouting

To illustrate the functional utility of this approach in a biological setting, PEG/HA hydrogels were applied in a 3-dimensional *in vitro* angiogenesis model and the cell-instructive role of HA was analyzed.

It was assessed whether PEG/HA hydrogels could support the formation of capillary-like sprouts in a routinely used spheroid sprouting assay. Therefore, spheroids from human umbilical vein endothelial cells (HUVECs) were embedded in degradable 3.5 w/v% hydrogels and the cumulative sprout length originating from each spheroid was quantified. Hydrogels with higher PEG polymer concentration and non-degradable PEG-dithiol crosslinker have been identified as inapplicable for the spheroid-sprouting assay, as the hydrogel stiffness and degradability constrained sprouting of HUVECs (see Supplementary Figure 3). A life/dead staining assay of evenly distributed HUVECs shows > 80% cell viability at day 2 (Supplementary Figure 3).

**FIGURE 6:**
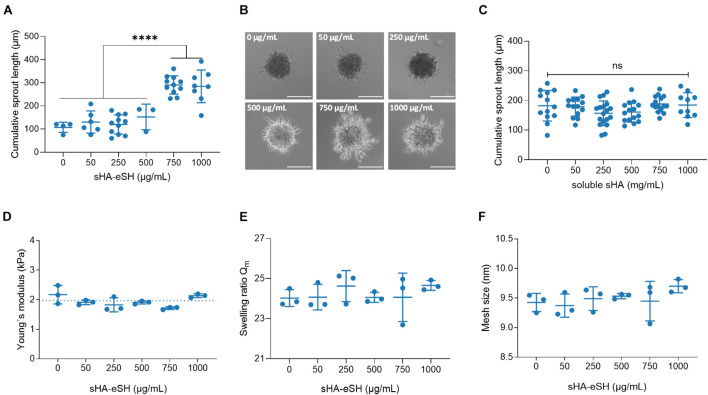
Influence of immobilized sHA on angiogenic sprouting. **(A)** Analysis of the cumulative sprout length originating from HUVEC spheroids, embedded in 3.5 w/v% PEG hydrogel functionalized with 0.5 mM linRGD and varying concentrations of sHA-eSH. Spheroids were stimulated with 50 ng/mL VEGF for 48 h. Statistical significance was calculated using one-way ANOVA followed by Tukey’s test (^****^p < 0.0001, *n* ≥ 3). **(B)** Representative phase-contrast images of VEGF-induced sprouting of spheroids embedded in PEG/HA hydrogels. Scale bar 50 μm. **(C)** Analysis of the cumulative sprout length originating from HUVEC spheroids, embedded in 3.5 w/v% PEG hydrogel functionalized with 0.5 mM linRGD and incubated with varying concentrations of freely diffusing sHA. **(D)** Bulk elasticity determined by shear rheological measurements, **(E)** swelling ratio of hydrogels in PBS and **(F)** mesh size of degradable 3.5 w/v% hydrogels prepared by thiol-Michael addition reaction with a MMP-cleavable di-cysteine peptide and functionalized with 0.5 mM linRGD and varying concentrations of sHA-eSH; mean and standard deviation of triplicates are presented.

Evaluation of six peptide motifs, derived from ECM proteins, revealed the highest sprouting capacity for the linRGD binding motif in degradable 3.5 w/v% hydrogels (Supplementary Figure 4). Thus, spheroids were embedded in degradable 3.5 w/v% PEG/HA hydrogels functionalized with varying physiological concentrations of sHA-eSH (0–1 mg/mL), while the linRGD concentration was maintained constant at 0.5 mM. Cumulative sprout length analyses reveal an increased sprout formation when spheroids are enclosed by hydrogels containing more than 0.75 mg/mL sHA-eSH (Figure 6A). Overall, HUVECs, invading into PEG/HA hydrogels with increasing sHA-eSH concentrations, seem to form sprouts with more branched and bulkier structures (Figure 6B). As previously, an analysis of the elasticity, swelling ratio and mesh size of degradable PEG/HA hydrogels revealed a stable network structure, unaffected by the immobilization of up to 1 mg/mL sHA-eSH (Figures 6D–F). Additionally, a presentation of immobilized HA, as part of an artificial ECM was compared to freely diffusing HA in soluble form. This time, the cumulative sprout length remained unaffected by the increasing concentration of soluble sHA (Figure 6C). These findings suggest that hyaluronan may enhance endothelial cell invasion in the presence of covalently immobilized sHA, while the presence of soluble sHA does not affect this behavior. These data demonstrate that the PEG/GAG hydrogels provide a chemically defined environment for the culture of endothelial cells and thus could overcome current existing systems by recapitulating the protein and GAG content of the ECM.

## Discussion

As key components of the ECM, GAGs mediate tissue hydration, structural scaffolding, local presentation of soluble molecules and cell signaling. Despite the increasing knowledge of GAGs and their bioactivity, the importance of their molecular weight, chemical structure and organization within the ECM is not completely understood. To systematically dissect the bioactive role of GAGs, we established a versatile biomaterial for a natural presentation of GAGs in a well-defined 3D environment.

For this purpose, previously end-thiolated GAGs were conjugated to a PEG hydrogel, formed by thiol-Michael addition reaction between a vinyl sulfone 8-arm PEG and a thiol containing crosslinker (Figure 1). We demonstrated the successful terminal modification of varying GAGs (heparin, chondroitin sulfate, dermatan sulfate, hyaluronic acid) and their immobilization to a PEG hydrogel. Diffusion experiments with fluorescently-labeled hyaluronan proved the necessity of conjugation to the backbone when the mesh size exceeds the radius of the GAGs (Figure 2). This GAG-conjugation approach provides a reproducible and defined presentation of desired GAG types in physiological relevant concentrations. The sulfated GAG content from diverse biological samples and decellularized ECMs is in the lower range of μg/mg tissue with an often tissue- and cell type-specific GAG expression (Barbosa et al., 2003; Wolf et al., 2012; Ventura et al., 2019). The successful quantification of sHA in swollen hydrogels (Figure 3) revealed efficient incorporation within the physiological range of the human body (e.g., 200 μg/g for large intestine and heart, 500 μg/g in the dermis) (Stern and Maibach, 2008; Cowman et al., 2015). Importantly, the PEG/GAG hydrogels introduced here require only a slight change at the reducing-end of the GAG polymer chain, while most developed GAG-based biomaterials yet exploit a crosslinking between GAG chains, which requires side-modifications of the repeating structure (Burdick and Prestwich, 2011; Broguiere et al., 2016; Ornell et al., 2019; Limasale et al., 2020). In general, modifications allow the incorporation of new features, but also critically compromise the chemical structure and change the specificity of protein-GAG interactions (Kjellén and Lindahl, 2018; Shi et al., 2021). To address this shortcoming for studying the GAG bioactivity, a terminal GAG immobilization strategy was established herein, which enables a natural presentation of GAGs within a 3D model while their backbone structure is conserved. Dynamic modifications (e.g., acetylation, sulfation, epimerization) of the GAG backbone produce a tremendous heterogeneity and guide interactions with soluble GAG-binding signaling molecules (Muthana et al., 2012). Sulfated GAGs modulate the trafficking, signaling activity and stability of growth factors and cytokines due to mainly electrostatic interactions with the highly anionic GAGs (Proudfoot et al., 2017; Köwitsch et al., 2018; Hachim et al., 2019). Using a hydrogel system in which heparin (derivatives) are crosslinked with 4-arm PEG, the group around Uwe Freudenberg was able to show that the heparin concentration and the sulfation pattern at heparin derivatives control the spatiotemporal availability of soluble growth factors (Limasale et al., 2020). This type of hydrogel is very successful for studying the controlled administration of soluble signaling molecules and their biological activity (Chwalek et al., 2014; Freudenberg et al., 2019). The herein presented terminal GAG immobilization strategy mimics the natural occurring conjugation of GAGs to proteins, and enables a comparison between a GAG-containing network and its GAG-free counterpart. Research focusing on growth factor-independent effects of GAGs (e.g.,CD44-HA interactions) and their biological implications, might benefit from an interaction with an unmodified GAG chain. Terminally modified GAGs are often applied to study specific GAG-protein interactions in QCM-D studies (Wolny et al., 2010; Dyer et al., 2017; Bano et al., 2018). In a previous study a reduced HA-protein interaction was detected for side-modified HA in comparison to terminally conjugated HA (Minsky et al., 2016). Furthermore, circumventing the use of side-modified GAGs for the formation of PEG/GAG hydrogels offers an uncoupling of the network structure from the presence of GAGs, which could not be achieved in exiting systems yet. Herein, HA concentrations in PEG/HA hydrogels were varied between 0 and 1 mg/mL at constant soft mechanical properties. Even higher HA concentrations are possible, although it must be considered that increasing consumption of PEG-VS arms might alter the network structure. Thereby, changes in storage modulus, gelation and swelling ratio upon high degrees of modifications are less profound for the 8-arm PEG-VS than for 4-arm PEG systems, as demonstrated by Kim et al. (2016).

Detailed analysis of the PEG/HA network structure revealed that elasticity, swelling behavior and network defects are controlled by the PEG polymer concentration, but not by the total HA content (up to 1 mg/mL sHA-eSH, Figure 4). In line with comparable PEG hydrogels (Kim et al., 2016; Rezakhani et al., 2020), designed for decoupling of the mechanical and biochemical properties, the PEG polymer concentration dictates the swelling ratio and stiffness. Tuning the stiffness in this manner mimics variances in stiffness of the ECM and are within the range of physiological elasticity of soft tissues (Engler et al., 2006; Chaudhuri et al., 2020). Moreover, constant thiol concentrations measured in an Ellman’s assay suggest an unaltered network formation upon functionalization with bioactive ligands. Increased crosslinking efficiencies were observed for a higher PEG concentration. Structural defects in end-linked networks mainly appear at lower PEG polymer concentrations, due to entanglements and intramolecular crosslinking and contribute to the determination of macroscopic properties (Lutolf and Hubbell, 2003). Recently, Rezakhani et al. (2020) accomplished networks with minimal structural defects at low solid content by rearranging the building blocks and the formation process. Hence, applying this alternative approach facilitates the formation of thiol-Michael addition hydrogels with lower solid content and higher bioactive ligand concentrations, beneficial for advanced 3D cell culture applications.

As a variety of biological processes require a continuous remodeling of the natural ECM, hydrogels are often composed of PEG macromers crosslinked with a matrix-metalloprotease (MMP)-cleavable di-cysteine peptide via a thiol-Michael addition reaction (Lutolf et al., 2003a). Upon higher portions of the MMP-cleavable peptide linker, reduced swelling ratios with limited impact on the elasticity was observed, suggesting an altered hydrophobicity of the polymer backbone and thus swelling behavior (Ebara, 2014). Degradation characteristics revealed that the degradability of the hydrogels could be controlled by the ratio of degradable peptide linker to non-degradable PEG-dithiol linker (Figure 5).

Next, we applied this highly controlled PEG/GAG hydrogel system to analyze the impact of the GAG hyaluronan on endothelial cell behavior. The essential process of angiogenesis, defined as the sprouting of new blood vessels from existing vasculature, occurs during development and wound healing (Saharinen et al., 2004; Potente et al., 2011). Its dysregulation is linked to several diseases, including cancer or ischemic and inflammatory diseases (Rajabi and Mousa, 2017; Fallah et al., 2019). The interaction of hyaluronan to its main receptor CD44 triggers specific cell signaling networks and promotes cell migration, proliferation and tube formation of endothelial cells (EC) (Chen et al., 2020).

Our analysis revealed that HA may enhance endothelial cell invasion in the presence of covalently immobilized sHA, while the presence of soluble sHA was not affecting this behavior (Figure 6). This presentation-dependent effect of hyaluronan (soluble vs. immobilized) is in line with previous studies, which state that the form of HA presentation alters CD44/RHAMM expression and co-localization, CD44-mediated cell adhesion and downstream signaling, contributing to an aggressive and invasive tumorigenic phenotype (Herrera-Gayol and Jothy, 2001; Sapudom et al., 2017; Amorim et al., 2018; Carvalho et al., 2021). Under physiological conditions, the spatial freedom of HA is limited by its structural organization within the ECM and pericellular coat (Toole, 2004; Wolf and Kumar, 2019). Despite the known importance of the structural role, most of the studies supplement the cell culture medium with HA and thus examine HA in its soluble form. Cultivation of endothelial cells in gelatin/HA hydrogels or on HA coated surfaces revealed enhanced cell motility, robust cytoskeleton formation and altered CD44 expression (Khanmohammadi et al., 2017; Pang et al., 2019). Next to the mode of presentation, the molecular weight (MW) of HA determines the complex physicochemical and biological properties of HA and its effects on angiogenesis and endothelial cell function. As previously demonstrated, high-MW HA restricts endothelial cell migration and proliferation *in vitro* and *in vivo* (Deed et al., 1997). Although fragments between 3 kDa and 20 kDa were first reported to induce angiogenesis, oligomers with 6–25 disaccharide units (<10 kDa) seem to have the most profound effects on endothelial cell proliferation, migration and tube formation and neovascularization *in vivo* (West et al., 1985; Slevin et al., 2002; Pardue et al., 2008; Gao et al., 2010; Wang et al., 2011). As sHA within these experiments has a MW of around 13 kDa, the immobilization of even smaller oligo-HAs is of high interest. In particular, 6–18-mers of HA (3–8 kDa) bind CD44 monovalent and thus impede CD44 clustering and alter downstream signaling (Amorim et al., 2018). In this context, it would be conceivable that immobilization of sHA leads to steric hindrance of CD44 binding and thus is sensed by the cell as shorter HA, explaining the different effects between immobilized and soluble forms detected in the PEG/HA hydrogels. Since HA effects on endothelial cells are numerous and dependent on the mode of presentation and the MW of each chain, the established PEG/GAG biomaterial might help to elucidate the context-dependent roles in more detail.

Furthermore, synergistic effects of VEGF and oligo-HA stimulation promote angiogenesis *in vitro* (Montesano et al., 1996) and result in a higher microvessel density upon implanting VEGF-loaded HA-hydrogels *in vivo* (Peattie et al., 2004). This effect was partly attributed to the availability of HA as a sequestration site, mimicking a native ECM environment to protect growth factors from degradation, thereby enriching their local concentration and mediating cell receptor interactions. In contrast, a recent study on GAG/VEGF_165_ interactions demonstrated that VEGF_165_ binding and EC sprouting behavior was unaffected by HA, while the sulfated derivatives were stimulating the sprouting of HUVEC spheroids in a sulfation-dependent manner (Koehler et al., 2019). Moreover, the authors revealed growth-factor independent effects of GAGs and suggest a GAG-mediated change in expression of proteins, associated with cell adhesion and cell signaling.

## Conclusion

In sum, end-thiolated GAGs can be conjugated in a highly controlled manner to the otherwise inert synthetic PEG polymer. Previous approaches regard the conjugation of bioactive peptides that facilitate cell adhesion or proteolytic degradation (Hern and Hubbell, 1998; Gibas and Janik, 2010; Cruz-Acuña et al., 2017). As GAGs interact with a variety of mediator proteins and modulate their biological activity, the PEG/GAG hydrogels introduced here, finally recapitulate a feature of the ECM environment systematically. Recent synthetic matrices, such as PEG hydrogels crosslinked by thiol-Michael addition reactions are chemically defined and highly reproducible in contrast to reconstituted basement membrane extracts (Aisenbrey and Murphy, 2020). Moreover, their customized physical or biochemical properties govern specific biological outcomes, e.g., beneficial for an organoid formation with structural and functional similarity to native tissues (Cruz-Acuña et al., 2017; Rezakhani et al., 2020). The presented end-thiolated GAG immobilization strategy provides a readily accessible method to vary the combination and concentration of GAGs, while it preserves precise control over the ligand density, enzymatic cleavage and mechanical properties of the matrices. The PEG/GAG hydrogels might contribute to quantitatively unravel the role of GAGs in directing cell fate and provide a bioactive scaffold for advanced 3D cell culture, opening new applications in disease modeling, drug screening and tissue engineering.

## Data Availability Statement

The raw data supporting the conclusions of this article will be made available by the authors, without undue reservation.

## Author Contributions

CZ designed and executed experiments. PM and HB assisted in data collection and provided advice on experimental design. HB and CZ wrote the manuscript. All authors reviewed and commented on the manuscript.

## Conflict of Interest

The authors declare that the research was conducted in the absence of any commercial or financial relationships that could be construed as a potential conflict of interest.

## Publisher’s Note

All claims expressed in this article are solely those of the authors and do not necessarily represent those of their affiliated organizations, or those of the publisher, the editors and the reviewers. Any product that may be evaluated in this article, or claim that may be made by its manufacturer, is not guaranteed or endorsed by the publisher.
