# Supplementary material for: Natural Presentation of Glycosaminoglycans in Synthetic Matrices for 3D Angiogenesis Models
Source: Front Cell Dev Biol. 2021 Oct 4;9:729670. doi: 10.3389/fcell.2021.729670 (PMC8521059; doi:10.3389/fcell.2021.729670)
Supplement: Supplementary file 1 [file Data_Sheet_1.docx]

Supplementary Material


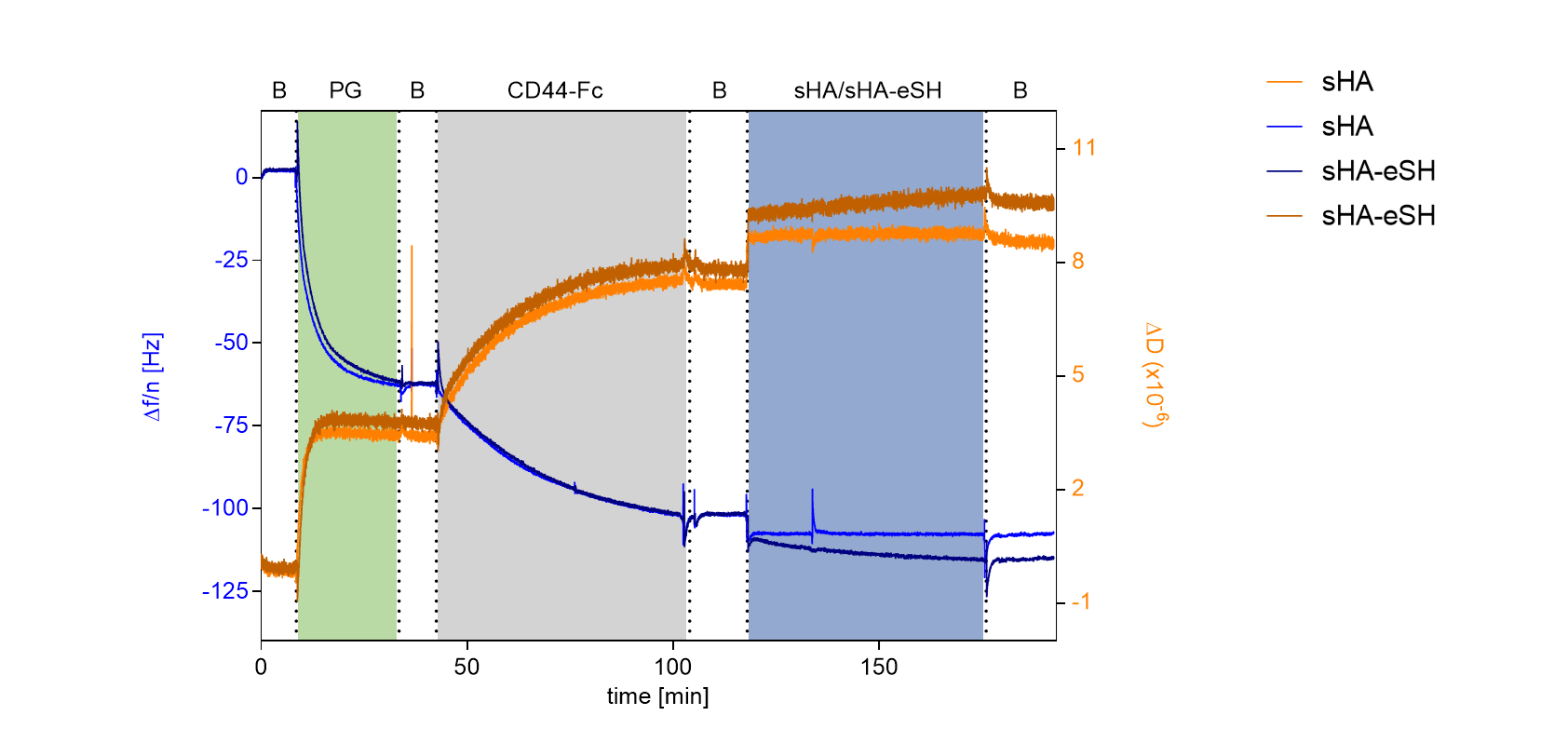


**Supplementary Figure 1.** QCM-D measurements confirm CD44 interactions with hyaluronan. The binding experiment shows the formation of a stable adlayer of protein G (PG) on a gold surface, followed by immobilization of Fc-tagged CD44 and its binding to sHA and sHA-eSH. Data from the 7^th^ overtone are shown, frequency changes depicted in blue and dissipation changes in orange. As physiological conditions are used for this experiment, the change in frequency observed upon sHA-eSH binding is twice as high as for sHA, probably due to disulfide bond formation. This indicates that sHA-eSH binds to CD44 with the same effectivity.


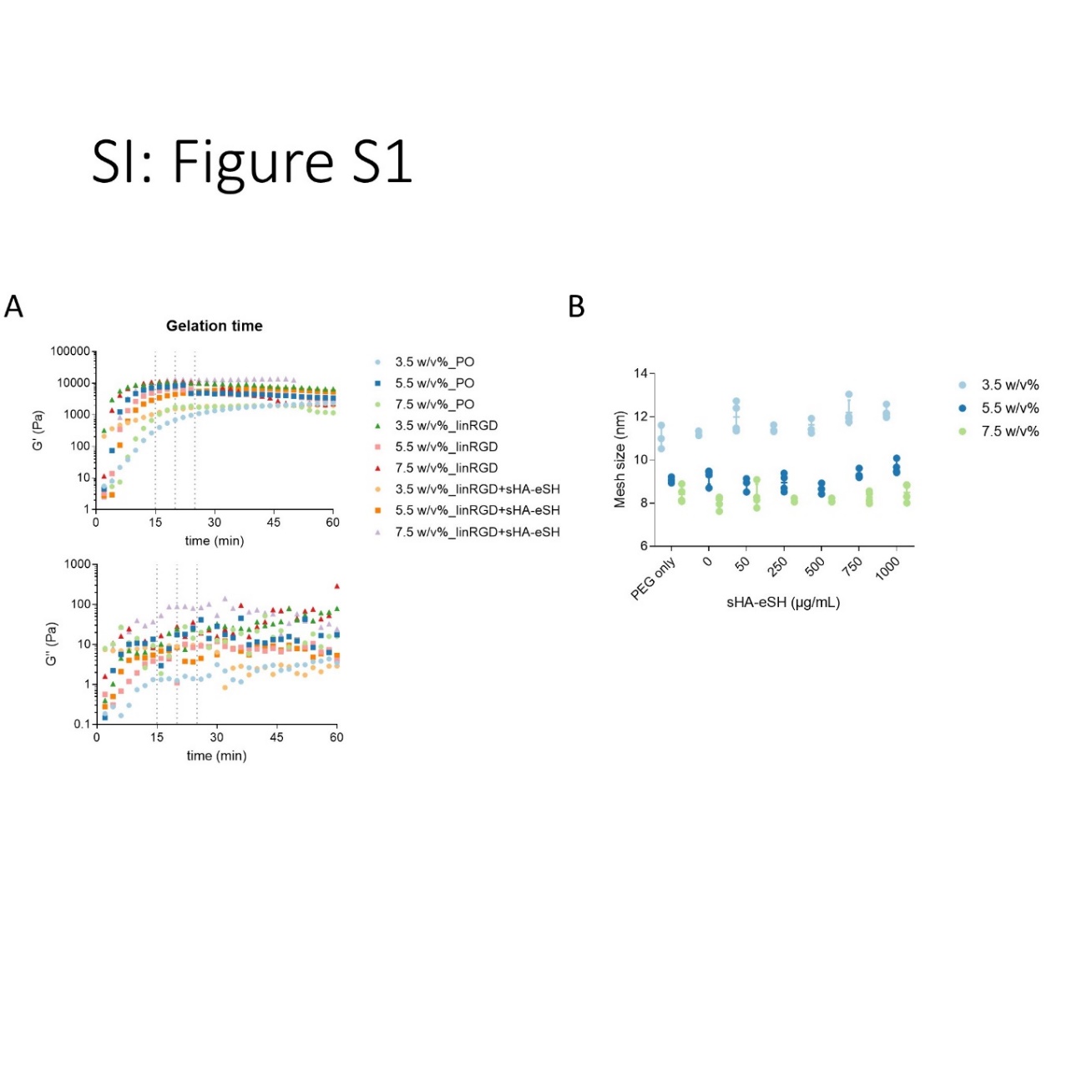


**Supplementary Figure 2.** (**A**) Gelation times obtained by time sweep experiments. Changes in the storage modulus G′ (Pa) and loss modulus G′′ (Pa) during hydrogel formation were monitored under a constant shear rate at 37 °C. (**B**) Mesh sizes, determined from swelling ratios in PBS. PEG hydrogels with three different polymer concentrations and degrees of functionalization (0.5x10^-3^ M linRGD and varying concentrations of end-thiolated sHA) were prepared by thiol-Michael addition reaction. Hydrogels were crosslinked with the PEG-dithiol linker. PO = PEG only, non-functionalized.


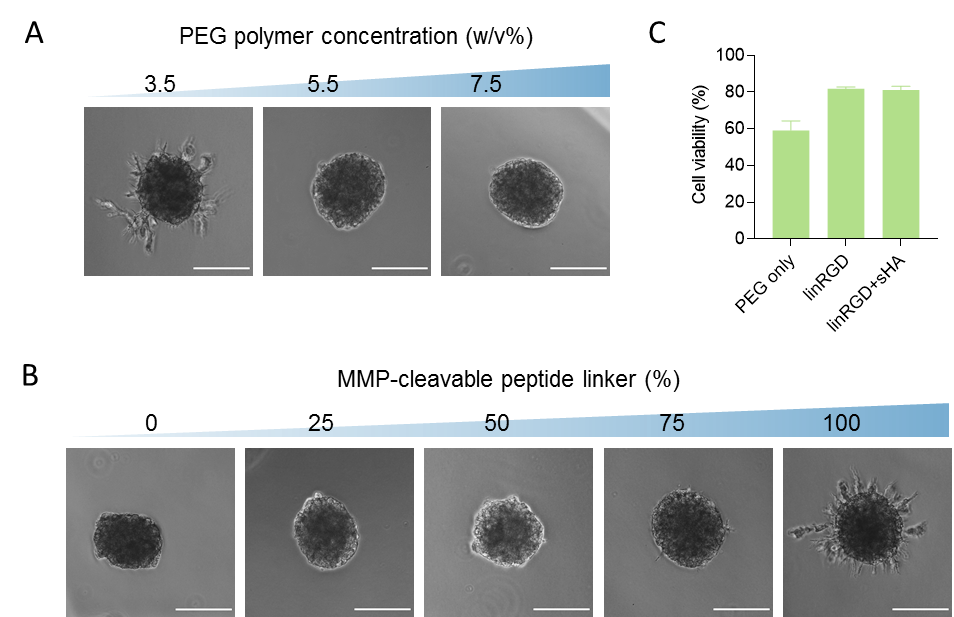


**Supplementary Figure 3.** PEG polymer concentration and enzymatic degradability constrains endothelial sprouting. Representative phase-contrast images of endothelial cell sprouts originating from PEG hydrogel embedded HUVEC spheroids. (**A**) Degradable hydrogels were prepared with three different polymer concentrations (3.5 w/v%, 5.5 w/v% and 7.5 w/v%) by Michael-type addition with a MMP-cleavable di-cysteine peptide. (**B**) 3.5 w/v% hydrogels were prepared by Michael-type addition reaction and crosslinked with a mixture of a non-degradable PEG-dithiol and a MMP-cleavable di-cysteine peptide in varying molar ratios. Percent refers to the ratio of MMP-cleavable peptide. Embedded HUVEC spheroids were stimulated with 50 ng/mL VEGF for 48 h. Scale bar 50 µm. (**C**) life/dead assay of evenly distributed HUVECs reveals 80% cell viability at day 2 of cultivation in degradable 3.5 w/v% hydrogels functionalized different bioactive cues.

**
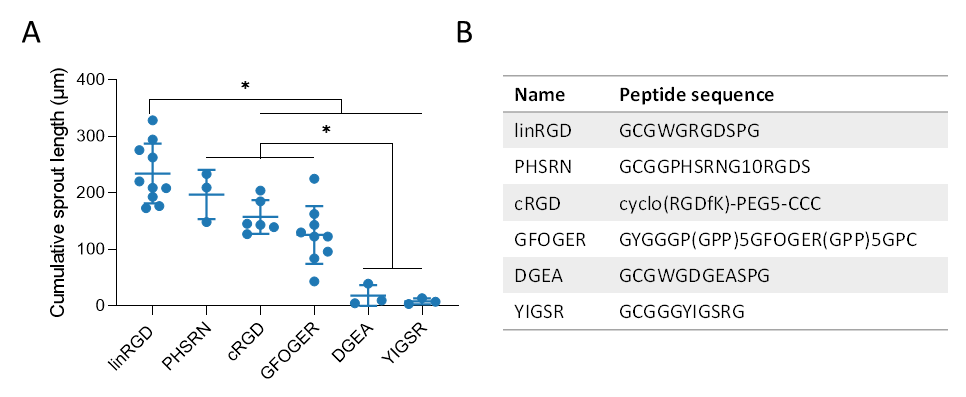
**

**Supplementary Figure 4.** Evaluation of six different peptide binding motifs on sprouting of endothelial cells. (**A**) Cumulative sprout length originating from HUVEC spheroids, embedded in 3.5 w/v% PEG hydrogel functionalized with 1x10^-3^ M peptide binding motif. HUVECs were stimulated with 50x10^-9^ g mL^-1^ VEGF for 48 h. Statistical significance was calculated using one-way ANOVA followed by Tukey’s test (* p < 0.05). (**B**) Peptide sequences of tested binding motifs.


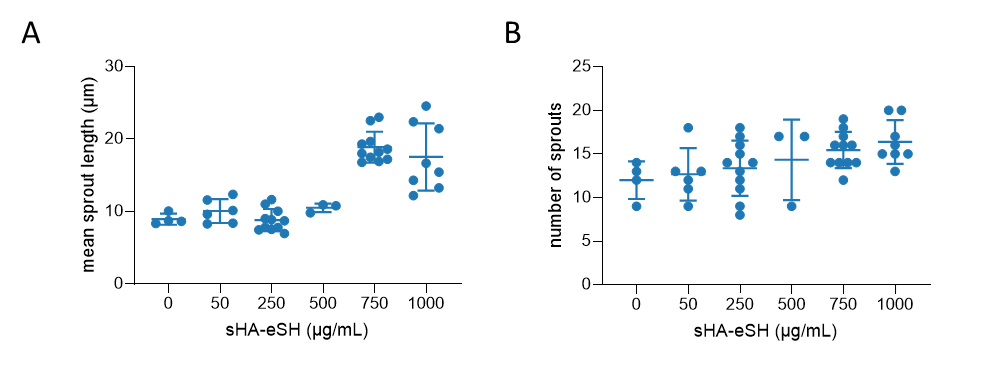


**Supplementary Figure 5.** Analysis of the (**A**) mean sprout length and (**B**) number of sprouts per HUVEC spheroid embedded in 3.5 w/v% PEG hydrogel functionalized with 0.5 mM linRGD and varying concentrations of sHA-eSH. Spheroids were stimulated with 50 ng/mL VEGF for 48 h.

**Supplementary Table 1.** Crosslinker concentrations (in mM) for hydrogel formation (exact concentration depends on the degree of functionalization with linRGD and sHA-eSH, as VS and SH groups are in a 1:1 molar ratio)

| Crosslinker | Functionalization | 3.5 w/v% | 5.5 w/v% | 7.5 w/v% |
| --- | --- | --- | --- | --- |
| Non-degradable | PEG only | 11.7 | 18.3 | 25.0 |
|  | 500 µM linRGD | 11.3 | 17.9 | 24.6 |
|  | 500 µM linRGD + 76.9 µM sHA-eSH | 11.2 | 17.9 | 24.5 |
| Semi-degradable* | PEG only | 11.0 | 17.3 | 23.6 |
|  | 500 µM linRGD | 10.6 | 16.9 | 23.2 |
|  | 500 µM linRGD + 76.9 µM sHA-eSH | 10.5 | 16.8 | 23.1 |
| Degradable | PEG only | 10.4 | 16.3 | 22.3 |
|  | 500 µM linRGD | 10.0 | 16.0 | 21.9 |
|  | 500 µM linRGD + 76.9 µM sHA-eSH | 10.0 | 15.9 | 21.9 |

* 1:1 molar ratio of non-degradable PEG-SH crosslinker and MMP-cleavable peptide crosslinker

**Example: Hydrogel calculations.**

Example calculation for 100 µL non-degradable 3.5 w/v% hydrogel, functionalized with 500 µM linRGD and 1 mg/mL (76.9 µM) sHA-eSH:

🡺 1. PEG-VS and PEG-SH make up 3.5 w/v% = 35 mg/mL (3.5 mg in 100 µL)

🡺 2. VS and SH functional groups at equal stoichiometric ratio (1:1)

|  | Number of functional groups per molecule | MW (g/mol) | Mass ratio for 1:1 molar ratio of SH:VS functional groups |
| --- | --- | --- | --- |
| PEG-SH | 2 | 1,000 | 8 x 1,000 Da = 8,000 Da – 1 part |
| PEG-VS | 8 | 20,000 | 2 x 20,000 Da = 40,000 Da – 5 parts |

1) How many SH groups are introduced by the functionalization with 500 µM linRGD and 76.9 µM sHA-eSH?

linRGD: 100 µL x 500 µM = 50 nmol

sHA-eSH: 100 µL x 76.9 µM = 7.69 nmol

- In total 57.69 nmol SH groups

2) How much 8-arm PEG-VS is consumed by the functionalization with linRGD and sHA-eSH?

SH groups = VS groups = 57.69 nmol

(57.69 nmol x 20,000 Da)/8 = 0.144 mg

- The mass of PEG-VS, which is needed for the conjugation of linRGD and sHA-eSH is 0.144 mg (mass_PEG-VS for functionalization_)

3) Which mass of PEG-VS and PEG-SH are additionally needed for a 3.5 w/v% hydrogel?

Total PEG in 100 µL: 3.5 mg

3.5 mg = mass_PEG-VS for functionalization_ + mass_PEG-VS_ + mass_PEG-SH_

- mass_PEG-VS_ + mass_PEG-SH_  = 3.5 mg – 0.144 mg = 3.356 mg

4) How is the mass ratio for formation of hydrogels with an equal VS:SH molar ratio?

The mass ratio is 1:5 PEG-SH: PEG-VS for having equal molar ratios of SH:VS groups.

mass_PEG-SH_: 3.356/6 x 1 = 0.559 mg (1118.40 nmol)

mass_PEG-VS_: 3.356/6 x 5 = 2.796 mg (1176.09 nmol)

🡺 mass_PEG-SH_ + mass_PEG-VS_ + mass_PEG-VS for functionalization_ = 0.559 mg + 2.796 mg + 0.144 mg = 3.5 mg

🡺 molar ratios SH (linRGD, sHA-eSH + PEG-SH): VS (PEG-VS) 🡺 (57.69 nmol +1118.40 nmol):1176.09 nmol or (0.049+0.9519):1 or 1:1
